# Pelvic tilt and stiffness of the muscles stabilising the lumbo-pelvic-hip (LPH) complex in tensiomyography examination

**DOI:** 10.1371/journal.pone.0312480

**Published:** 2024-10-23

**Authors:** Karol Bibrowicz, Tomasz Szurmik, Piotr Kurzeja, Bartosz Bibrowicz, Katarzyna Ogrodzka-Ciechanowicz

**Affiliations:** 1 Science and Research Center of Body Posture, Kazimiera Milanowska College of Education and Therapy, Poznań, Poland; 2 Faculty of Arts and Educational Science, University of Silesia, Cieszyn, Poland; 3 Institute of Health Sciences, University of Applied Sciences in Nowy Targ, Nowy Targ, Poland; 4 Research and Development Center Legia Lab, Legia Warszawa, Warszawa, Poland; 5 Faculty of Motor Rehabilitation, Institute of Clinical Rehabilitation, University of Physical Education, Krakow, Poland; Brunel University London, UNITED KINGDOM OF GREAT BRITAIN AND NORTHERN IRELAND

## Abstract

**Purpose:**

The objective of the study was to initially validate the hypothesis about the relationship between the pelvic tilt angle in the saggital plane and the functional state of muscles stabilising the lumbo-pelvic-hip (LPH) complex expressed as a change in their stiffness in a tensiomyography examination.

**Materials and methods:**

Forty five women aged 19–30 years took part in an observational (cross-sectional) study. The examination involved measurements using the tensiomyography method (TMG). The stiffness of muscles stabilising the LPH complex expressed as a maximal muscle displacement (Dm variable) was assessed and the relationship between muscle stiffness and the value of the pelvic tilt (PT) in the sagittal plane was determined.

**Results:**

The analysis showed significant differences in the values of medians of the muscle displacement (Dm) values in groups identified in terms of the value of pelvic tilt (Table 1) for Erector Spinae (ES) muscles (p = 0.0012), Gluteus Maximus (GM) muscles (p = 0.0004), Rectus Abdominis (RA) muscles (p = 0.0005), Obliquus abdominis externus (OAE) muscles (p = 0.0002*) and Rectus Femoris (RF) muscles (p = 0.0071). The results of the correlation analysis performed using the Spearman rho correlation coefficient between the value of pelvic tilt and muscle stiffness (Dm) show the following significant relations for ES muscles (p = 0<0.0001), GM muscles (p<0.0001), RA muscles (p<0.0001) and OAE muscles (p<0.0001). However, a clear direction of changes in stiffness in accordance with the description of relations defined as Lower Crossed Syndrome was not confirmed.

**Conclusions:**

A tensiomyographic examination did not show clear relations between the value of pelvic tilt and stiffness of muscles stabilising the lumbar-pelvic-hip complex. The mechanism of Lower Crossed Syndrome (LCS) may be not the only model explaining the relations between musculofascial structures of the hip-lumbar area. The implications of the LCS should not be the only basis for the therapy of disorders resulting from an incorrect position of the pelvis in the sagittal plane.

## Introduction

In physiotherapy the assessment of pelvic tilt in the sagittal plane is an important element of the whole process of examining static body posture and a functional condition of the locomotive organ. This is related to the stabilising and buffering role of the pelvis [1]. Changes in its position in the sagittal plane may be of significance in dysfunction in postural control, back pain, problems with hip and knee joints [2–7]. A clinical assessment of the pelvic tilt usually involves the measurement of the angle between the line connecting superior iliac spines, anterior and posterior, and a horizontal line [7,8]. For the measurement of the pelvic tilt in the sagittal place, as well as in the frontal and transverse planes, various types of inclinometers [7–10], levels built in smartphones [11] or ultrasonographic examinations are used [12]. The reliability and repeatability of the results obtained with their use is high and the results can be comparable with the results of radiography [10,11,13–16]. When describing the occurrence of increased values of the pelvic tilt (PT) in the sagittal plane, a reference is often made to a model of functional imbalance of muscles stabilising the lumbar-pelvic-hip complex (LPH complex) described by Vladimir Janda as a Lower Crossed Syndrome (LCD) [17]. It is related to the shortening and increased tone of tonic muscles, e.g. hip flexors and lumbar extensors, as well as the stretching and reduced tone of phasic muscles, e.g. abdominal muscles and gluteal muscles [18,19]. Sedentary lifestyle [20,21] and being overweight [22], among other things, are cited as reasons. The concept of muscle tone and muscle stiffness is therefore a multi-faceted concept.

The assessment of muscle tone is one of the basic elements of diagnostic tests of the musculoskeletal system. We can talk about changes in the tone of skeletal muscles in dysfunctions of the upper or lower motor neuron [23], about stiffness being a determinant of the muscle elongation capacity (passive stiffness) [24], or the consequences of muscle fatigue following physical exertion [25]. To assess the changes in muscle tone and muscle stiffness palpation can be used [26,27], as well as special clinical tests for selected muscle groups [24] and devices specially developed to assess muscle tone and stiffness [27]. The literature concerned with the attempts to quantify the changes in muscle tone lists methods based on electromyography [28], myotonometry [29,30], shear wave elastography [31] or magnetic resonance elastography [32]. In this context the concept of tensiomyography (TMG) also appears frequently in scientific literature. TMG is an non-invasive measurement method used to detect properties of skeletal muscles. It was developed in 1983 by professor Vojko Valenčič. Initially, it was used only in medicine, however since 1996 it has been also successfully applied in sport [16,33–35]. TMG makes it possible to perform a comprehensive functional assessment of skeletal muscles based on the analysis of their reaction to a set electric stimulus [29]. One of the aspects of the assessment of the functional state of muscles is the assessment of their stiffness [36,37]. One of the best documented parameters in the literature obtained in a tensiomyographic examination is maximal displacement (Dm) assessing the size of radial displacement of the belly of a muscle following a stimulation with an electric stimulus and indicating muscle stiffness [36,38,39].

In scientific literature the problem of increased or decreased pelvic tilt in the sagittal plane is described quite well. Similarly, much is known about clinical consequences related to incorrect values of the pelvic tilt. We also note a development of various therapeutic concepts aiming to restore the balance within the muscles stabilising the pelvis in order to bring it to its correct position [4,19,28,43,45,55,56]. Most often they are based, to a greater or lesser degree, on the concept of muscular imbalance developed by Janda. Literature notes also certain doubts whether Janda’s model is optimal for the therapy of disorders related to an incorrect pelvic tilt in the sagittal plane. The purpose of this study arises also from these doubts. We wanted to verify whether changes in stiffness of muscles stabilising the LPH complex are related to the changes in the pelvis position in the sagittal plane and whether the LCS concept according to Janda can be confirmed in the light of the tensiomyographic examinations.

The objective of the study was to initially validate the hypothesis concerning the relationship between the value of pelvic tilt in the sagittal plane and the functional state of the muscles stabilising the lumbar-pelvic-hip complex, as expressed by a change in their stiffness in tensiomyography studies.

## Material and methods

### Study design

This is an observational cross-sectional study. The study protocol follows the guidelines of the Helsinki Declaration and was conducted in compliance with the Strengthening the Reporting of Observational Studies in Epidemiology (STROBE) Statement: guidelines for reporting observational studies [40].

The observation was conducted as part of a long-term research program approved by the Bioethics Committee of the College of Physiotherapy in Wrocław (No. 1/2010, 22.12.2010).

### Setting

The study was carried out between March and November 2020 at the College of Education and Therapy in Poznań, Poland.

### Participants

176 female physiotherapy students of the College of Education and Therapy in Poznań, Poland, were qualified for the study. The survey revealed that none of the women surveyed were engaged in competitive sport.

Inclusion criteria:

a/ Age between 19 and 30 years, b/ Right lateralisation of the hand and foot, c/ No discernible anomalies were observed in the subject’s body composition. d/ Body mass Index (BMI) between 18,5–24,9.e/ Symmetrical pelvis. (A symmetrical pelvic position was defined as a condition in which the line joining the tops of the iliac plates, the anterior superior iliac spines and the great vertebrae is situated in a parallel orientation to the ground [9].

Exclusion criteria:

a/ Pain in the lumbo-pelvic-hip complex area or use of analgesics during the tests and two weeks prior to the examination, b/ pregnancy or menstrual phase.

From the studied group 45 women meeting the inclusion and exclusion criteria were selected in a simple random selection, in three sub-groups of 15 persons each, formed on the basis of the value of pelvic tilt determined in accordance with the author’s own clinical typology [8]:

1. 15 women with a decreased pelvic tilt–PT < 12°
2. 15 women with a normal pelvic tilt–PT ≥ 12 ≤ 20°
3. 15 women with an increased pelvic tilt–PT > 20°

### Outcome measures

The examination involved measurements using the tensiomyography method. According to the methodology of tensiomyography only muscles in the superficial group may be analysed. In the study the muscles stabilising the lumbar-pelvic-hip complex were selected for the analysis. The following muscles were examined: gluteus maximus (GM), biceps femoris (BF), rectus femoris (RF), rectus abdominis (RA), abdominal external oblique muscle (OAE), spinal erector muscle (ES).

**Fig 1:**
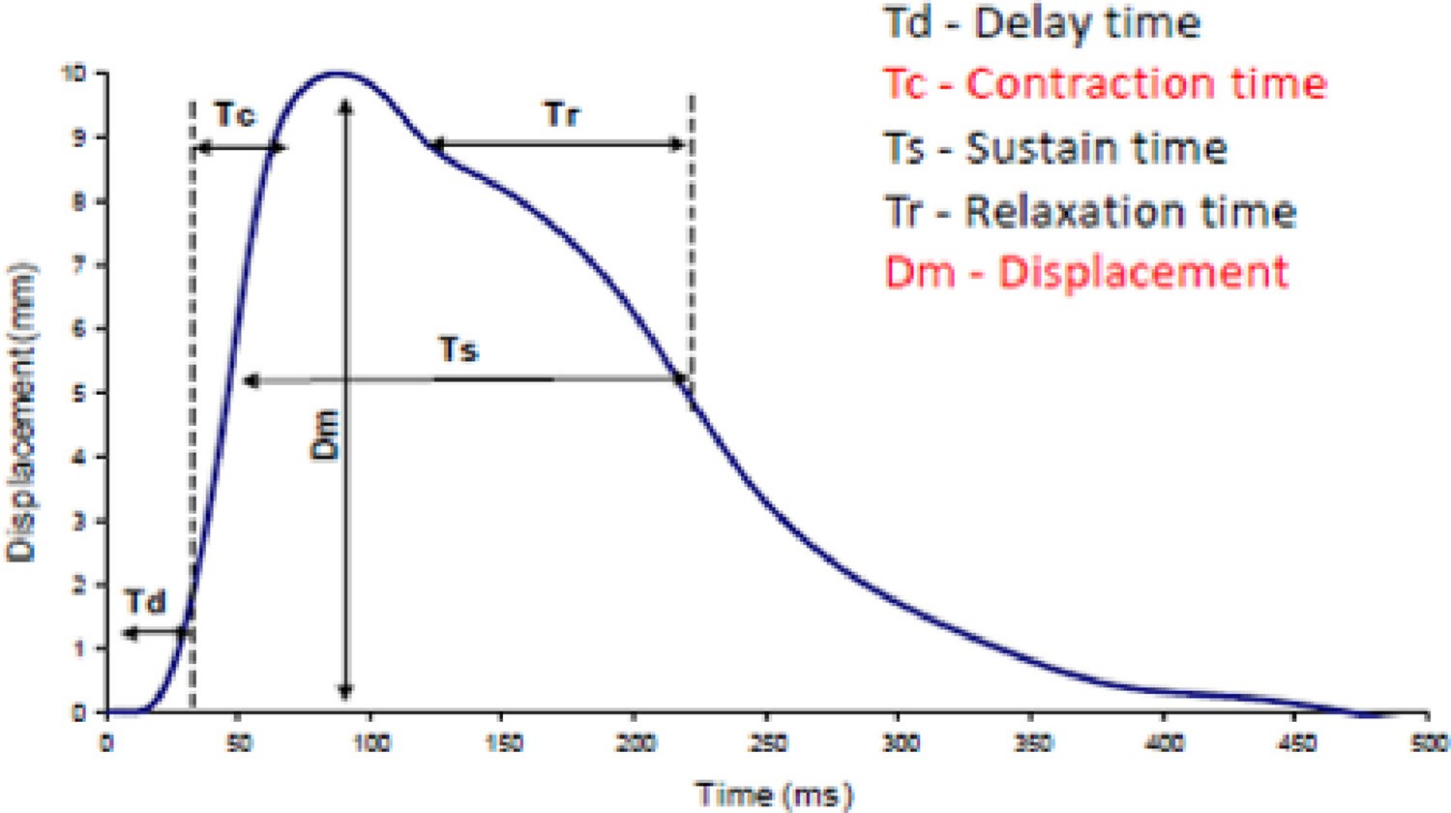
An example of a diagram of a muscle reaction to an electric stimulus [own source].

One of the parameters available in the tensiomyography method describing the functional state of muscles–Dm (maximal muscle displacement measured in millimetres [mm])–illustrating the size of radial displacement of the belly of the muscle and its stiffness [38,39,41] was selected for detailed analysis (Fig 1).

For each of the study participants a measurement of pelvic tilt using a Duometer© was also performed.

### Assessment

All tests were performed in the morning, ensuring that uniform conditions of taking measurements are maintained. Measurements were performed three times for each person and the mean data were recorded in the protocol. All inclinometric and tensiomyographic measurements were carried out by the same, experienced examiner.

#### Pelvic tilt inclinometry

Pelvic tilt (PT) was measured in a relaxed standing position, defined as the angle between the level and the line running through the apices of the anterior and posterior superior iliac spine.

**Fig 2:**
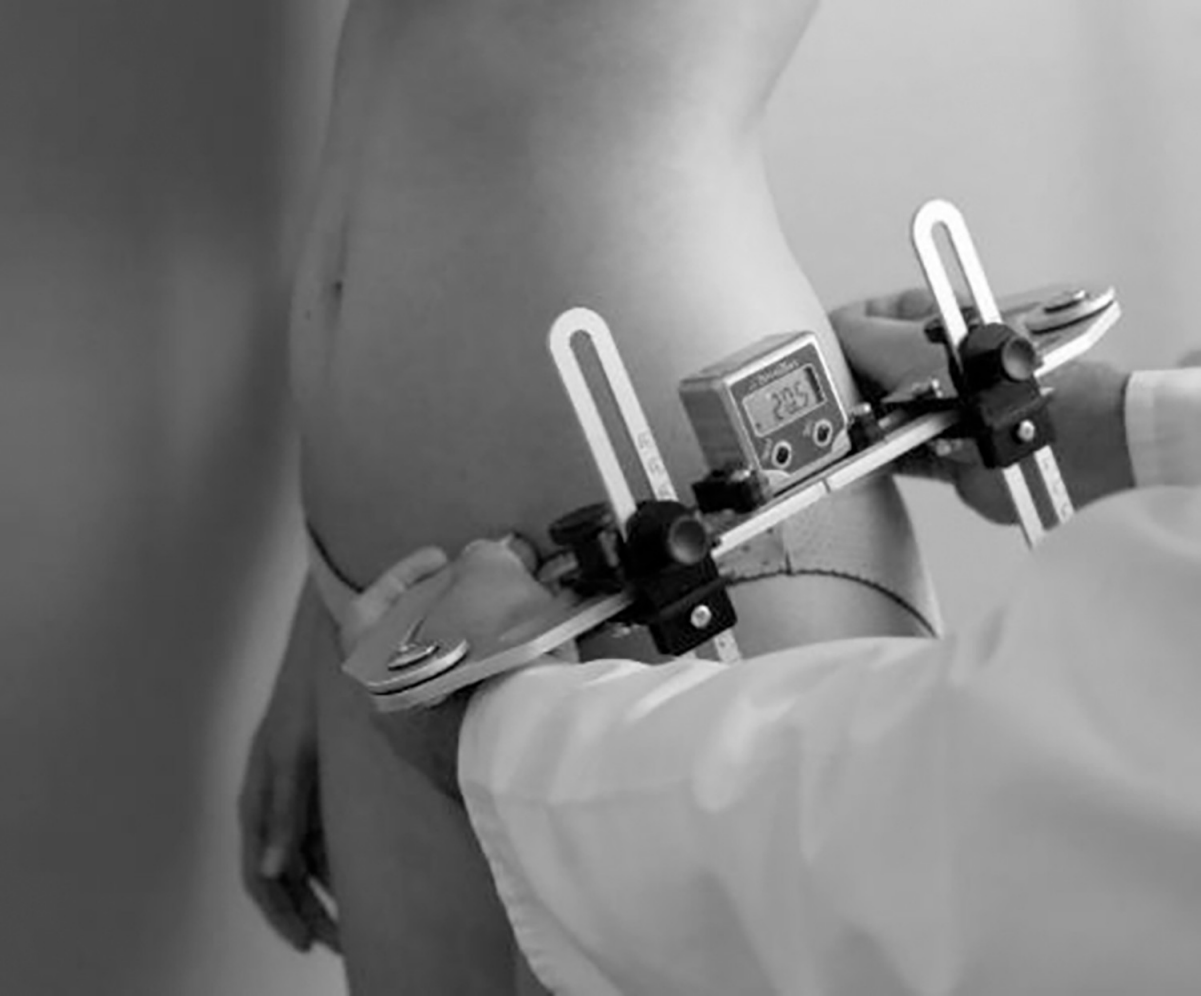
Measurement of pelvic tilt using a Duometer © [own source].

The examination in the sagittal plane was performed according to the author’s methodology, by combining palpation and inclinometry, and was performed by the same experienced examiner. The measurements of position of the pelvic girdle in the sagittal plane were taken on the subjects’ left sides [8]. During the examination the principle was observed that the examined bone point was touched with the tip of the middle finger and the arms of the measuring device were placed strictly on the radial side of the middle finger (Fig 2).

After meeting the inclusion criteria, 45 women divided into three groups of 15, in accordance with the clinical typology of pelvis, participated in the study. In each case a written, conscious consent was obtained from the participants to include them in the study.

#### Tensiomyography test (TMG)

**Fig 3:**
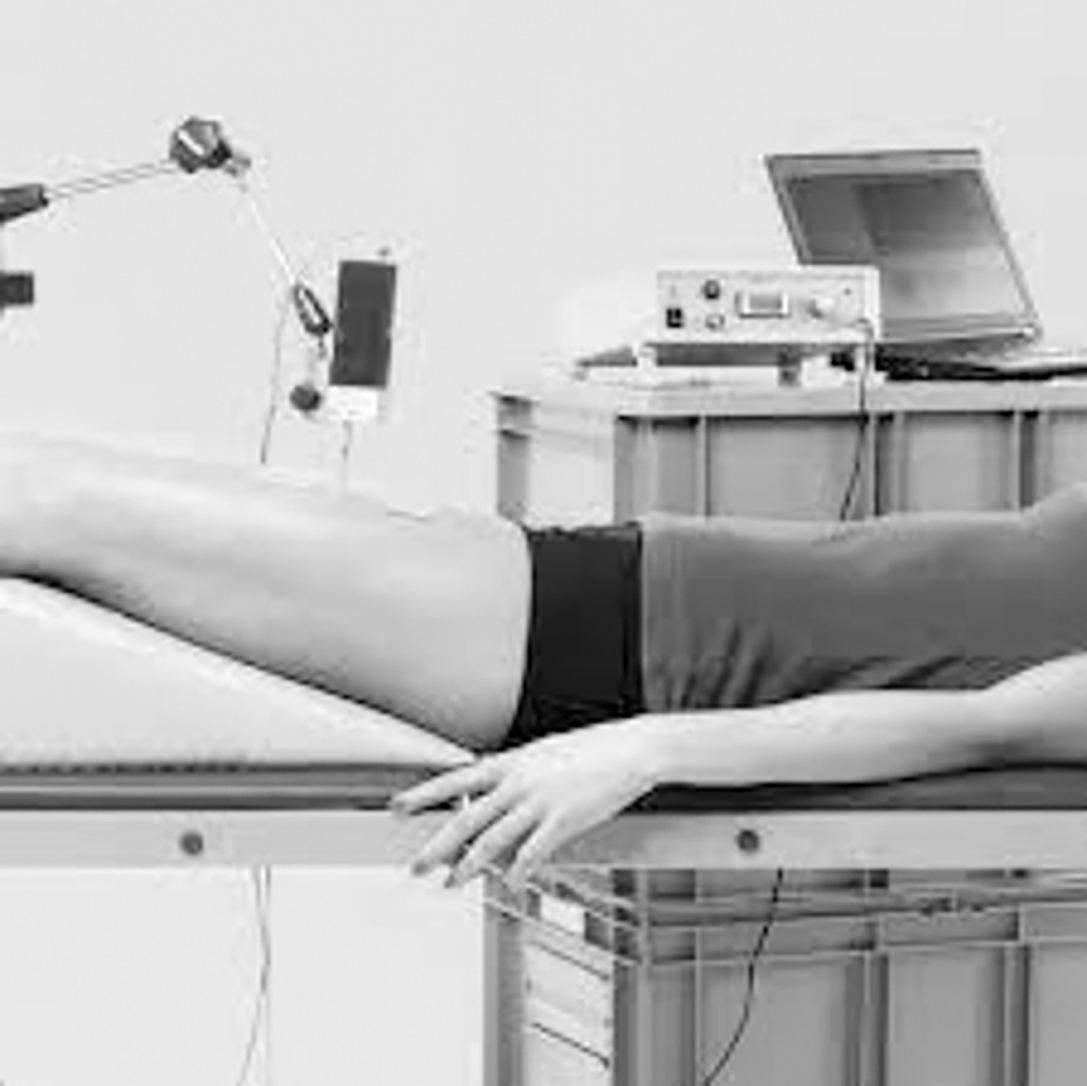
An example of a measurement of a rectus muscle of thigh [own source].

The TMG tests were carried out using a TMG^TM^ Science for Body Evolution system (TMG-BMC Ltd., Ljubljana, Slovenia). The methodology of measurement of individual muscles according to the procedures proposed by the device manufacturer was used. The placement of electrodes was in line with the SENIAM procedure [42]. All measurements were performed in a laboratory at the temperature of 22 ± 1°C. Measurements of ES, GM and BF muscles were carried out in a prone lying position with ankle joints placed on a triangular wedge bolster. RA, OAE and RF muscles were examined in the supine lying position with legs bent in knee joints at the 20° angle. A tensiomyographic examination involves the assessment of the muscle reaction (reaction time and size of displacement) to a set electrical stimulus. Two standard, self-adhesive ECOSTIM electrodes, 2 mm thick (EL. P.NWCS50.50.sq. Shiaoxing, China 5x5cm) were placed symmetrically, distally and proximally from the end of TMG sensor (2.5 cm in each direction). A device for TMG measurements (TMG-BMC Ltd., Ljubljana, Slovenia) was used in the examinations. Electrical stimulation was provided using a single square pulse of 1 ms, with an intensity set at 20 mA and increased by 10mA every 10–15 s until further change of Dm was noted or maximum stimulator power was achieved (110 mA). The cathode was placed proximally and the anode distally (Fig 3).

The values of displacement (Dm) describing muscle stiffness presented in the study, obtained using tensiomyographic method are mean values of measurements performed on the left and right side of the body.

### Statistical methods

The statistical analysis of the study material was performed using a MedCalc® Statistical Software version 22.023 (MedCalc Software Ltd, Ostend, Belgium). The assessment of distribution of variables was performed using the Shapiro-Wilk test. A standard descriptive analysis was presented using, depending on distribution characteristics, mean and standard deviations or median values (M) and interquartile range (IQ). The differences between anthropometric variables were calculated using the ANOVA analysis of variance. The differences in results of measurements of specific muscles in separate groups were calculated using the non-parametric Kruskal-Wallis test. In case of statistically significant differences the Conover post-hoc analysis was performed. Additionally, the trend analysis using the Jonckheere-Terpstra test was assessed. The strength of the relationship between the value of pelvic tilt and the studied tensiomyographic variables was calculated using Spearman’s (rho) rank correlation method.

## Results

After meeting the inclusion criteria, 45 women divided into three groups of 15, in accordance with the clinical typology of pelvis, participated in the study. In each case a written, conscious consent was obtained from the participants to include them in the study.

**Fig 4:**
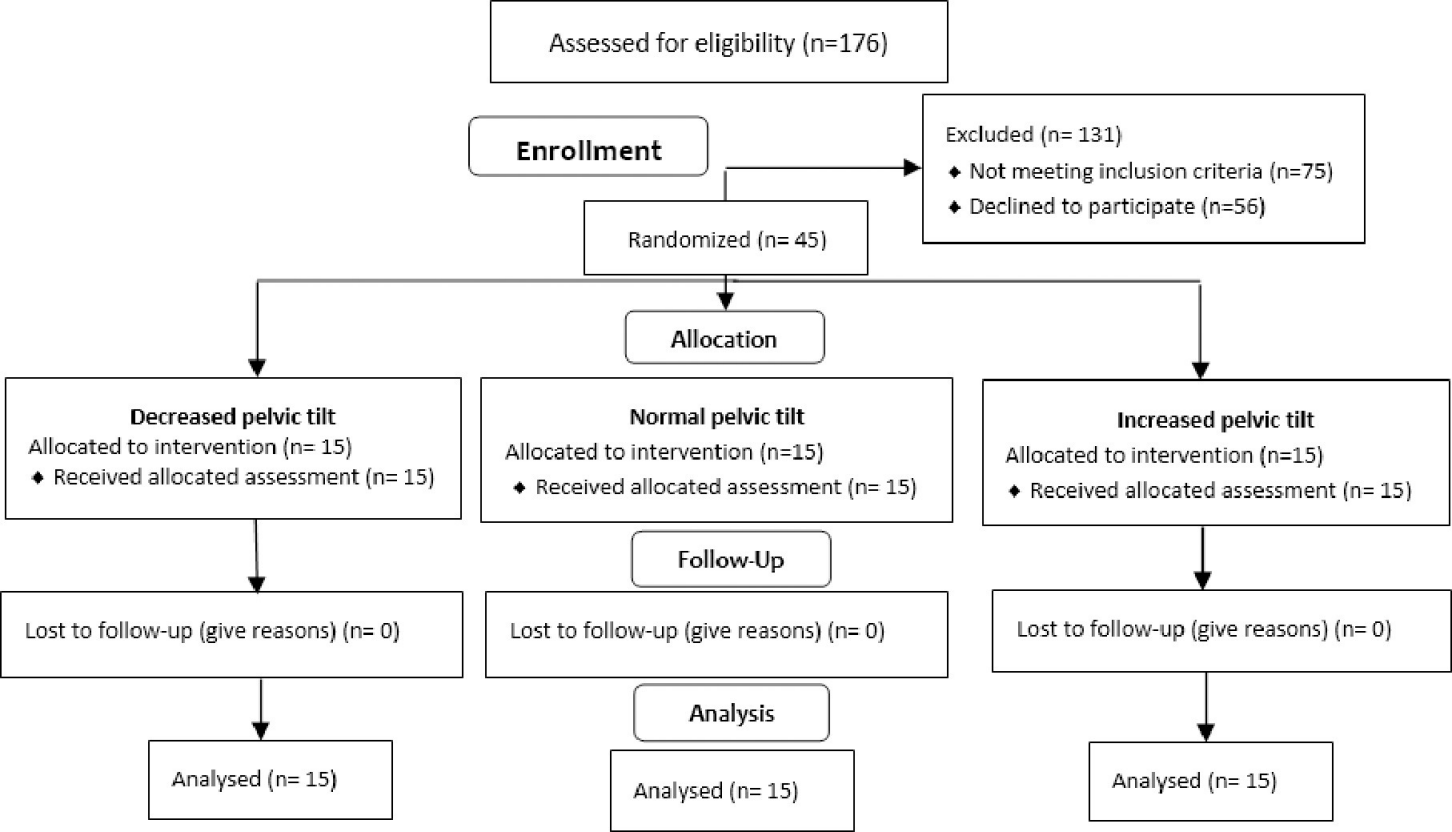
Flow diagram.

Table 1 Presents the detailed anthropometric data of the sample. Fig 4 shows the qualification stage.

**Table 1:** Study group.

Group Variable	Decreased pelvic tilt	Normal pelvic tilt	Increased pelvic tilt	ANOVA
	X ± SD	X ± SD	X ± SD	
**Age(yrs)**	23.4 ± 2.32	23.7 ± 3.19	22.2 ± 2.21	p = 0.453
**Body weight[kg]**	61.4 ± 4.56	57.9 ± 4.41	60.3 ± 5.51	p = 0.231
**Height [cm]**	166.4 ± 1.24	165.8 ± 6.67	167.3 ± 5.51	p = 0.706
**BMI [kgm-** ^**2**^ **]**	22.1 ± 3.21	21.4 ± 3.21	22.7 ± 3.21	p = 0.123

X–mean, SD–standard deviation.

The values of displacement (Dm) describing muscle stiffness presented in the study, obtained using tensiomyographic method are mean values of measurements performed on the left and right side of the body.

In the studied group the values of pelvic tilt measurements ranged from 10 to 26 degrees (X = 16.9, SD = 5.34). The results of tensiomyographic analysis of the studied muscles are presented in Table 2.

**Table 2:** Values of median (M), interquartile range (IQ) and the results of the analysis performed using the Kruskal-Wallis test for maximal muscle displacement (Dm) in groups formed based on the value of pelvic tilt.

Dm [mm]				
Group Muscle	Whole group	Decreased pelvic tilt	Normal pelvic tilt	Increased pelvic tilt
	M IQ	M IQ	M IQ	M IQ
**ES**	3.2 2.15–4.20	1.9 0.89–3.13	3.6 3.09–3.97	3.5 2.68–6.61
**GM**	6.1 2.23–7.55	5.9 2.23–6.61	4.6 1.4–6.27	8.2 6.08–10.37
**BF**	4.0 3.03–5.71	4.0 3.83–4.94	4.5 2.18–6.00	3.8 3.50–6.80
**RA**	3.4 2.57–5.26	2.7 1.48–2.87	3.7 3.01–4.66	4.6 3.24–5.87
**OAE**	1.8 0.96–3.32	1.4 0.96–1.48	1.6 0.65–3.2	3.2 2.29–3.55
**RF**	5.4 4.39–8.03	5.4 5.31–7.85	4.6 2.90–6.69	7.7 1.17–9.10

M–Median; IQ–Interquartile range; K-W–Kruskal-Wallis test; p – statistical significance; GM–Gluteus maximus; BF–Biceps Femoris; RF–Rectus Femoris; RA–Rectus Abdominis; OAE–Obliques Abdominis Ext; ES–Erector Spine.

The performed analysis showed significant differences in values of medians of displacement value (Dm) in groups formed on the basis of the pelvic tilt value (Table 3) for ES (p = 0.0012), GM (p = 0.0004), RA (p = 0.0005), OAE (p = 0.0002) and RF muscles (p = 0.0071). The assessment of differences in individual groups was carried out using the Conover post-hoc test. It showed that for ES muscles the differences were noted between the group with the decreased value of pelvic tilt compared to the groups with normal and increased pelvic tilt values. Similar differences were noted for RA muscles. For GM and OAE muscles differences were noted between the groups with decreased and increased pelvic tilt and between the groups with normal and increased pelvic tilt. For RF muscles the differences were noted between the group with normal pelvic tilt and the groups with decreased and increased pelvic tilt. The analysis of the trend performed using the Jonckheere-Terpstra test showed a significant trend between the value of pelvic tilt and the values of radial displacement of the belly of muscle for ES (p = 0.00060*), GM (p = 0.00493*), RA (p = 0.00009*) and OAE muscles (0.00015*).

**Table 3:** Analysis of correlation (Spearman’s rho) between the value of pelvic tilt (PT) and stiffness of muscles stabilising the LPH complex in the whole group.

Dm [mm]					
	ES	GM	BF	RA	OAE
**PT (degree)**	Rho = 0.430 p<0.001*	Rho = 0.427 p<0.0001*	Rho = 0.064 p = 0.5505	Rho = 0.505 p<0.0001*	Rho = 0.506 p<0.0001*

PT–pelvic tilt; Rho–Spearmann’s correlation index

*–statistical significance; GM–Gluteus maximus; BF–Biceps Femoris; RF–Rectus Femoris; RA–Rectus Abdominis; OAE–Obliques Abdominis Ext; ES–Erector Spine.

The results of the correlation analysis carried out using Spearman’s rho correlation coefficient between the value of pelvic tilt and muscle stiffness (Dm) showed such significant relations for ES (p = 0<0.0001)*, GM (p<0.0001*), RA (p<0.0001*) and OAE muscles (p<0.0001*) (Table 3).

As no clear linear relationship was found between the value of pelvic tilt (PT) and displacement value (Dm) for individual muscles in which statistically significant correlation was noted, it was decided that an additional analysis should be performed in groups formed on the basis of the value of pelvic tilt. The results are presented in Table 4.

**Table 4:** The assessment of relations between muscle displacement Dm (stiffness) and pelvic tilt (PT) in the studied groups.

Muscle Pelvis	ES	GM	RA	OAE
**Decreased** **pelvic tilt**	Rho = -0.381 P = 0.0002*	Rho = -0.128 p = 0.236	Rho = -0.392 p = 0.0001*	Rho = -0.342 p = 0.002*
**Normal** **pelvic tilt**	Rho = 0.130 p = 0.223	Rho = -0.292 p = 0.006*	Rho = 0.075 p = 0.480	Rho = -0.068 p = 0.540
**Increased** **pelvic tilt**	Rho = 0.252 p = 0.017*	Rho = 0.415 p = 0.001*	Rho = 0.317 p = 0.002*	Rho = 0.423 p = 0.0001*

*–statistical significance; GM–Gluteus maximus; BF–Biceps Femoris; RF–Rectus Femoris; RA–Rectus Abdominis; OAE–Obliques Abdominis Ext; ES–Erector Spine.

The analysis performed in this way showed that for women with a marked decreased pelvic tilt such significant relations were noted for ES, RA and OAE muscles. In all cases the values of correlation were negative, which means that the smaller the pelvic tilt, the greater the stiffness of the muscles. For women with normal values of pelvic tilt, significant relation was noted only for GM muscles and again it was a negative value. Different results were noted in the group with increased pelvic tilt. For all analysed muscles the correlations were significant and had positive values. This means that for ES, GM, RA and OAE muscles the stiffness of muscles decreased with the increase of pelvic tilt (Table 4).

## Discussion

The results of the own study do not provide a definite answer whether there is a relationship between changes in the pelvic tilt in the sagittal plane and stiffness of muscles stabilising the LPH complex. The analysis of results obtained for individual muscles does not allow for a definite confirmation of the expected behaviour of tonic and phasic muscles. For example, for RF muscles greater muscle stiffness with an increased pelvic tilt angle would be expected. However, greater stiffness of this muscle expressed as the value of maximal displacement (Dm) was paradoxically noted in the group with a decreased pelvic tilt angle compared to the group with an increased pelvic tilt angle. These differences were statistically significant. Also an unclear situation was noted for ES, the second muscle in the group of tonic muscles. In this case also statistically significant differences in the value of Dm were noted calculated in the groups formed on the basis of the value of pelvic tilt. In this case however, the greatest muscle stiffness was noted in the group with a decreased pelvic tilt angle. For phasic muscles, namely GM, BF, RA and OAE muscles, the picture is not clear either. For RA and OAE abdominal muscles the greatest stiffness was noted in groups with a decreased pelvic tilt angle, and the smallest stiffness was noted where the pelvic tilt was the greatest. For these muscles also statistically significant differences were noted between the studied groups. For the GM muscle the greatest stiffness was noted with the normal value of pelvic tilt, the smallest was noted in the group with increased pelvic tilt. The values of Dm for the BF muscle were noted with some surprise. Here, irrespective of the pelvic tilt value, the values of Dm were on a comparable level. No significant statistical differences in the studied Dm variable were noted for this muscle either.

The correlation analysis using Spearman’s rho rank correlation showed such a relation between muscle stiffness Dm and the value of pelvic tilt for ES, GM, RA and OAE muscles. For BF and RF muscles no such correlation was noted.

Different findings were reported by Kuszewski et al. who noted moderate, negative correlation for muscles of the posterior thigh (hamstring) between the pelvic tilt and muscle stiffness [43].

For a fuller picture of relationships between muscle stiffness and the value of pelvic tilt, a stratification analysis was carried out in groups formed on the basis of the value of pelvic tilt. Based on the obtained results, various levels of relations between Dm and PT were noted. In the group with a decreased pelvic tilt all studied correlations were negative, which indicates that the smaller the pelvic tilt, the greater the muscle stiffness. Correlations were however only statistically significant for ES, RA and OAE muscles. The results in the group with increased PT values were however different. Here all correlations were statistically significant and positive. This indicates that with an increase in pelvic tilt the values of Dm increased, which means that the muscle stiffness decreased.

The obtained results indicate that we can actually confirm that there are certain relations between the value of pelvic tilt and muscle stiffness expressed as values of Dm in a tensiomyographic examination. These relations are not as definite as it was assumed at the beginning of the study. However, they confirmed some findings made earlier by Van Gelder and Mills [44,45] that perhaps the model proposed by Janda does not describe the relations between the functional state of the muscles stabilising the pelvis and the value of pelvic tilt. Such a set of results may have a number of causes. Firstly, muscle stiffness is not a clear concept. It is not properly defined and is still an object of discussion and study.

Changeability of opinions and no clear definition of muscle tone mean that during an examination of muscle tone it is always necessary to clarify how this term should be understood [27,46]. Usually we deal with passive or dynamic stiffness [47]. In order to assess passive stiffness, apart from clinical tests, methods based on elastographic, myotonometric and tensiomyographic measurements are usually applied. In each of these methods different aspects related to tissue stiffness are differently examined and analysed which makes it difficult to compare the results [27,41,48,49]. In the TMG analysis stiffness is indirectly calculated and interpreted on the basis of maximum radial displacement (Dm) [37]. In myotonometry stiffness is defined differently, as a relation between the strength produced by a mechanical impulse and the depth of tissue deformation [50].

Irrespective of the definitions of this concept, changes in the length and stiffness of muscles stabilising the lumbar-pelvic-hip complex are often treated as a direct cause of excessive or insufficient pelvic tilt in the sagittal plane and the main direction of therapy. Mostly they are based to a greater or smaller extent on the concept of muscular imbalance proposed by Janda [18,19]. It seems well confirmed how incorrect pelvis position affects the locomotive system, both in the biomechanical context [7,51] and in terms of its clinical consequences [3–5,19,52,53]. Various forms of therapy are developed aiming to restore correct anatomical and biomechanical conditions within the lumbar-pelvic-hip complex, starting from the administration of various pharmacological measures [54] to various systems of exercise restoring correct tone and length of pelvis stabilisers [4,19,28,43,45,55,56].

A question however arises whether this is the optimal approach. Although it seems rational and we witness in therapeutic practice practical effects of the therapy based on such assumptions. According to the authors this is however an expression of a simplified approach to the problem. There are publications which note that the relation between the shortened and tensed hip flexors and the stretching and decreasing the tone of gluteal muscles and hamstring muscles is not so clear [44,45]. According to the authors, therapies focusing on the local approach to solving the problem of imbalance of the muscles stabilising the lumbar-pelvic-hip complex using the mechanism of reciprocal inhibition may be insufficient [57]. Perhaps the therapy of imbalance disorders should be extended to include the assessment of function of higher level mechanisms controlling the muscle tone at the level of the central nervous system, in particular pontomedullary reticular formation (PMRF) [58–60].

### Study limitation

The authors are aware that the most important limitation in determining the relations between muscle stiffness and the value of pelvic tilt is the fact that there is no proper definition of the concept of muscle tone and muscle stiffness. We can only note the presence or absence of the relation for pelvic tilt and muscle stiffness determined in a tensiomyographic examination as maximal displacement (Dm). For a clear relation between stiffness and PT the study would have to include other methods, assessing the stiffness in other aspects as well as the assessment of muscle strength and length. Another limitation also resulting from the applied methodology of study was the impossibility to reach deeper muscles, for example, iliopsoas muscles. Selecting only women for the study group could also present a limitation. Although, one of the inclusion criteria was no menstruation and no pregnancy, but changes in muscle stiffness may take place also in other phases of the menstrual cycle. To gain a more comprehensive understanding of the matter, it would be beneficial to expand the study group and conduct analyses according to additional factors, such as age, gender, physical activity, and the specific sport practised.

## Conclusions

A tensiomyographic examination did not show definite relations between the value of pelvic tilt and the stiffness of muscles stabilising the lumbar-pelvic-hip complex. The mechanism of lower crossed syndrome (LCS) may be not the only model explaining the relations between musculofascial structures of the hip and lumbar area. The implications of LCS should not be the only basis for the therapy of disorders resulting from the incorrect position of pelvis in the sagittal plane.

## Supporting information

S1 Data (XLSX)
